# The contribution of activity theory to modeling multi-actor decision-making: A focus on human capital investments

**DOI:** 10.3389/fpsyg.2022.997062

**Published:** 2022-09-27

**Authors:** Silvia Marocco, Alessandra Talamo

**Affiliations:** Department of Social and Developmental Psychology, Sapienza University of Rome, Rome, Italy

**Keywords:** decision-making, socio-cultural psychology, multi-actor, human capital investments, activity theory, startup funding

## Abstract

Making investment decisions is usually considered a challenging task for investors because it is a process based on risky, complex, and consequential choices (Shanmuganathan, 2020). When it comes to Investments in human capital (IHC), such as startups fundings, the aspect of decision-making (DM) becomes even more critical since the outcome of the DM process is not completely predictable. Indeed, it has to take into consideration the will, goals, and motivations of each human actor involved: those who invest as well as those who seek investments. We define this specific DM process as multi-actor DM (MADM) since not a group is making decisions but different actors, or groups of different actors, who – starting from non-coinciding objectives – need to reach a mutual agreement and converge toward a common goal for the success of the investment. This review aims to give insights on psychological contributions to the study of complex DM processes that deal with IHC to provide scholars and practitioners with a theoretical framework and a tool for describing the complex socio-ecological systems involved in the DM processes. For this purpose, we discuss in the paper how the third generation of activity theory (Leont’ev, 1974, 1978;Engeström, 1987, 2001) could be used as an appropriate model to explain the specificities of MADM construct, focusing on the particular case of startup funding. Design thinking techniques will be proposed as a methodology to create a bridge between different activity systems.

## Introduction

Making investment decisions is usually considered a challenging task for investors, because it is a process based on risky, complex, and consequential choices (Shanmuganathan, 2020). Investing in any business implies the involvement of multiple factors, both external and internal to the decision-maker. External factors include the company’s balance sheet, inflation, and prevailing interest rates (Sevdalis and Harvey, 2007; Oehler et al., 2018). Internal factors are mostly psychological and involve cognitive and affective levels (Statman, 2017), which influence the decision-making (DM) process. Moreover, investments may be classified into two categories: investment in the capital market, such as financial securities, bonds, stocks, or investment in human capital (IHC), such as startups fundings. In IHCs, the aspect of DM becomes even more critical, since different actors with varying behaviors and agencies are involved. For this reason, IHC cannot be considered a one-sided investment, but a mutual investment that implies a specific process of DM, a multi-actor DM (MADM). In fact, this kind of DM does not involve single individuals, neither a group of decision-makers belonging to the same social context, but different actors, or groups of different actors, who start from non-coinciding objectives and that, through a process of negotiation, should make their goals *compatible –* able to coexist *-*, *coordinable –* able to complement each other’s *-*, *and convergent –* able to come closer together *-*, to reach a rewarding and mutual agreement. Therefore, this review aims to orient researchers in this field toward psychological theories that may better help modeling IHC processes and provide tools to describe them. More specifically, we will explain the model analyzing the case of a startup funding.

### The traditional contribution of cognitive psychology to the study of financial DM

#### Classification and analysis of the literature

**Figure 1:**
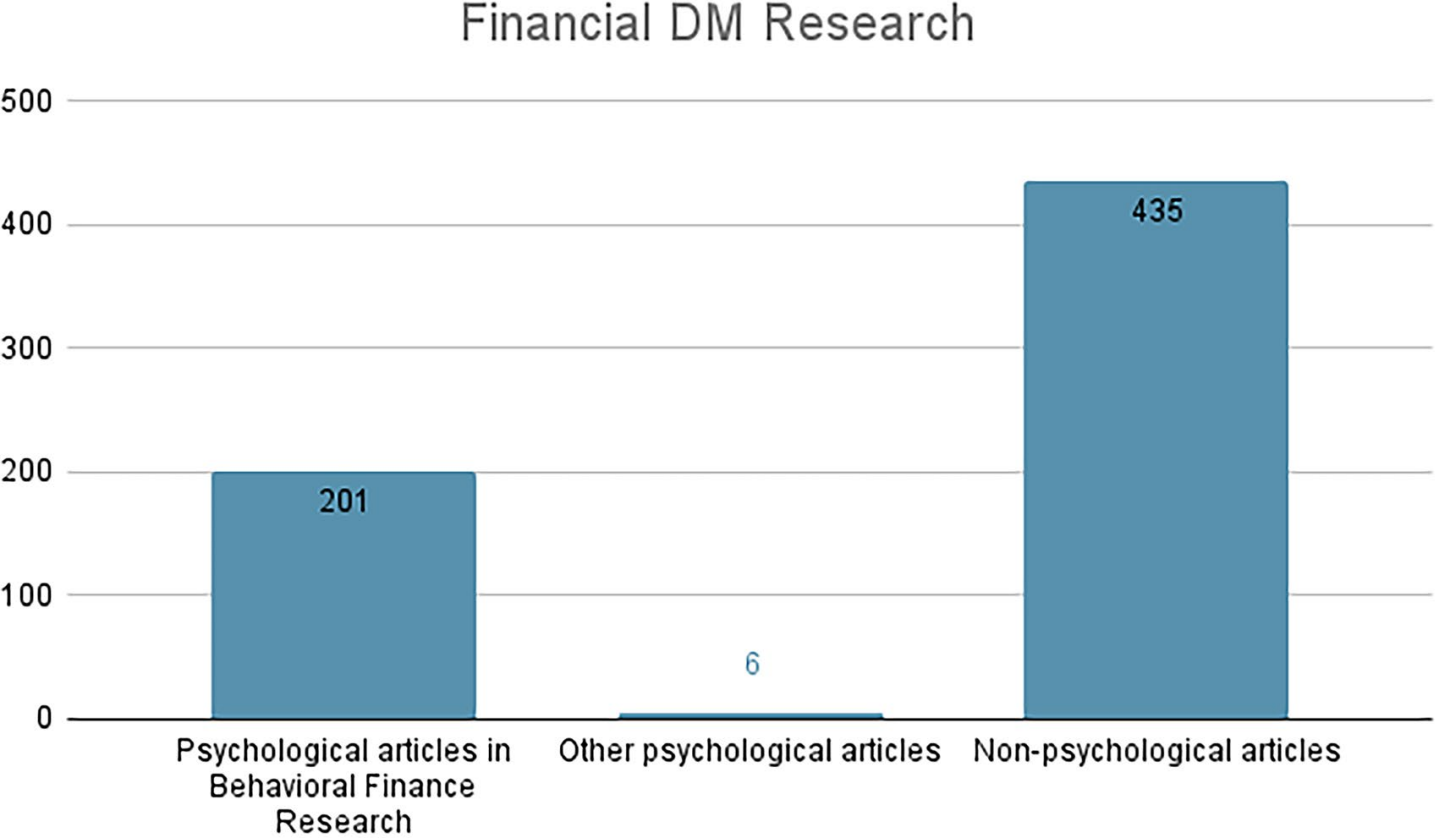
Classification of the eligible articles on FDM (*searched on Scopus on May 24*, *2022*).

Up to now, the greatest contribution of psychology to the study of financial DM (FDM) seems to come from the *Behavioral Finance* perspective, an interdisciplinary approach that includes scholars from the fields of Finance, Psychology (especially the branch of *Cognitive Psychology*^1^) and Sociology. This came out also from our literature analysis within the Scopus database. Specifically, the aim of our literature review was exploring how psychology has traditionally contributed to the study of FDM until now. To this purpose, we inserted the keywords *“financial”* and *“DM”* without any filters searching within *article titles*, *abstract and keywords*. Preliminary research identified 35,511 papers, showing how widely studied and debated this theme is. To carry out further screening, we entered the keywords only by searching for *article titles*. This research identified 655 papers. Then, we uploaded the Scopus database on *Rayyan*, an Intelligent Research Tool, in order to optimize the papers’ coding and selection. In total, 13 articles were deleted after the duplicate detection. In the end, the eligible articles (642) were coded into 3 classes (see Figure 1):

- *Psychological articles in Behavioral Finance Research* (201 articles; 31,3%): all those psychological articles aimed at contributing to Behavioral Finance research.
- *Other psychological articles on FDM* (6 articles; 0,9%): those articles that, although of a psychological nature, do not fit within the research trend of Behavioral Finance.
- *Non-psychological articles on FDM* (435 articles; 67,8%): all those articles belonging to other disciplines – such as computer science, mathematics, or engineering – that are not relevant for our purpose of investigation.

**Figure 2:**
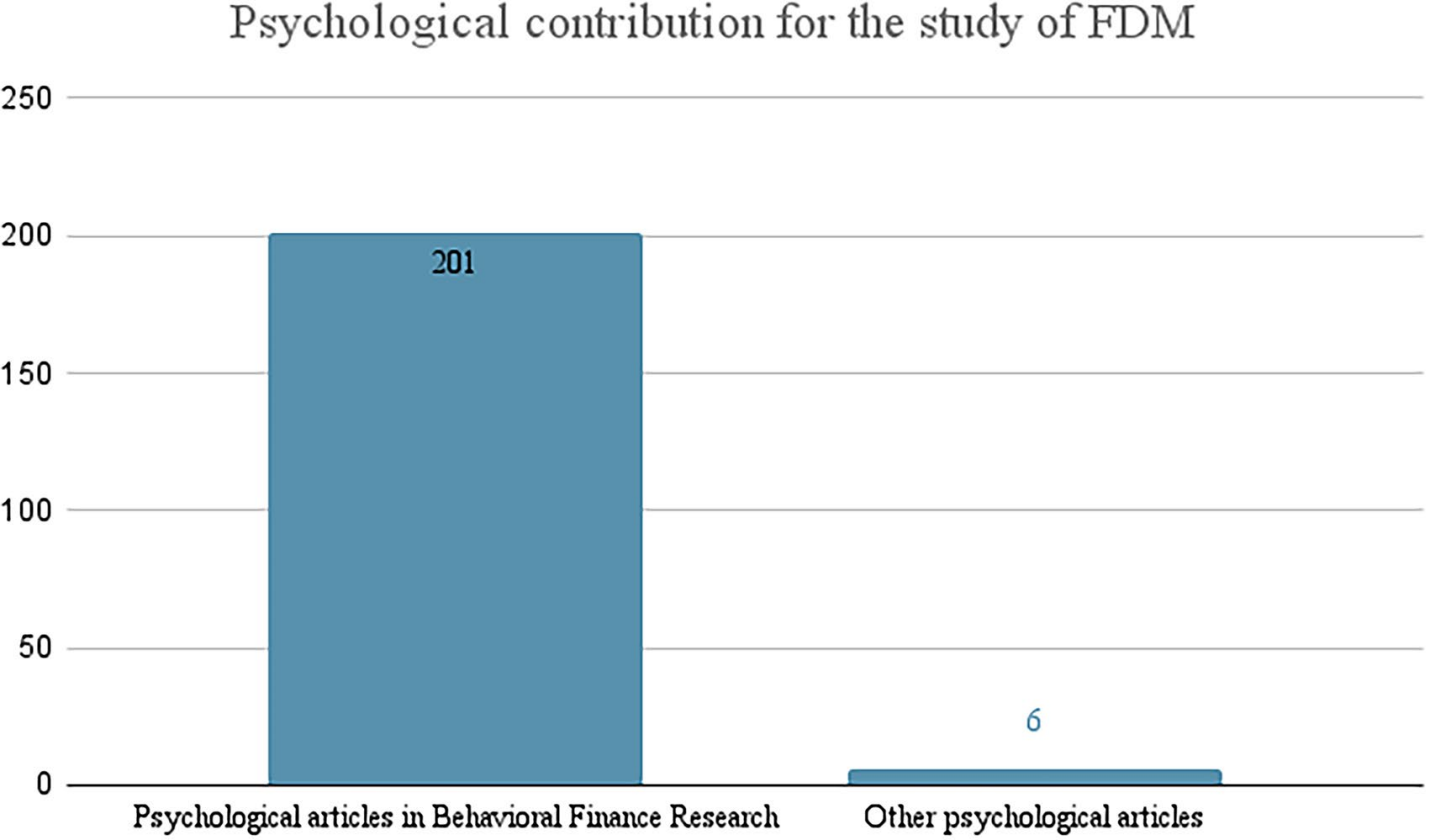
Classification of psychological articles on FDM (*searched on Scopus on May 24*, *2022*).

Hence, considering only the psychological articles (Figure 2), it comes out as evidence that the psychological contribution to the study of financial decisions, except for a very small part (6 articles; 2.9%), is aimed almost exclusively at the Behavioral Finance research line (201 articles; 97.1%).

Given the clear predominance of this approach to the study of FDM, for the purposes of our investigation it is worth asking: does this theoretical perspective offer a contribution also in the field of IHC? To answer this question, we must first understand what Behavioral Finance is and which are the key concepts that led it to its success.

#### The behavioral finance perspective: From rational to irrational individual FDM

The Behavioral Finance approach attempts to explain and increase the understanding of the reasoning patterns of investors, including the emotional processes involved and the degree to which they influence the DM process. *Essentially*, *behavioral finance attempts to explain the “what*, *why*, *and how” of finance and investing*, *from a human perspective* (Ricciardi and Simon, 2000). Researchers in this field argue that investors do not operate as fully rational decision-makers; instead, they are affected by psychological influences and biases that could drive them to make irrational investment decisions (Niehaus and Shrider, 2014).

According to Pompian (2006), a pioneering researcher of the field, Behavioral Finance (which, by many definitions, is included in Behavioral Economics) can be divided in two primary subtopics:

- *Behavioral Finance Micro* (BFMI) which examines *behaviors or biases of individual investors*, distinguishing them from the rational actors envisioned in neoclassical economics.^2^
- *Behavioral Finance Macro* (BFMA), which detects and describes *anomalies* in the efficient market hypothesis that behavioral models may explain.

One of the first investigators of BFMI was the economist and decision theorist Howard Raiffa, which in 1968 introduced to the decision analysis three approaches that provide a more accurate view of a “real” person’s decision process:

- *Normative* analysis, concerning the rational solution to the problem.
- *Descriptive* analysis, dealing with the way real people actually make decisions.
- *Prescriptive analysis*, focused on practical advice and tools that may help people obtain results closer to those of normative analysis.

#### The intellectual foundations of BFMI: Cognitive bias theory and prospect theory

Nevertheless, the most significant steps for the development of BFMI emerged from the result of *Cognitive Bias Theory* (Tversky and Kahneman, 1974) and *Prospect Theory* (Kahneman and Tversky, 1979), developed by both cognitive psychologists Daniel Kahneman and Amos Tversky during the 1970s. Their conceptualizations proved to be very helpful to economists for their attempt to model the way people actually make decisions instead of simply relying on the utility*^3^ DM strategies that had made up finance theory until then. Fundamentally, Tversky and Kahneman “*brought to light the incidence*, *causes*, *and effects of human error in economic reasoning”* (Pompian, 2006, p. 31).

More specifically, Tversky and Kahneman (1974) introduced the term *“cognitive bias”* to describe people’s systematic but purportedly flawed patterns of responses to judgment and decision problems under uncertainty (Wilke and Mata, 2012). According to them, these biases begin as the consequence of the use of heuristics or simple cognitive principles that decision-makers adopt to reduce cognitive or computational requirements (Gigerenzer et al., 1999). In this way, the “*Heuristics and Biases program*,” inspired by Herbert Simon (1956) principle of *bounded rationality*^4^, addressed the question of how people make decisions given their limited resources, due to cognitive limitations, motivational factors, and/or adaptations to natural environments (Wilke and Mata, 2012).

The other intellectual foundation of BFMI is *Prospect Theory*. This theory names two specific thought processes: *editing* and *evaluation*. During the editing state, alternatives are classified according to a basic *“rule of thumb”* (heuristic). Then, a reference point is designated during the evaluation phase, which provides a relative basis for evaluating gains and losses. More specifically, through this conceptualization, Kahneman and Tversky (1979) stated that, under conditions of uncertainty, people make decisions based on the potential value of gains and losses rather than the utility, and that loss makes a greater emotional impact on investors than gain (the tendency of *loss aversion*^5^). Richard Thaler, who was already a finance theorist at the time, perceived and manifested the necessity to apply Prospect Theory to financial markets, becoming, together with Tversky and Kahneman, one of the founding fathers of Behavioral Finance.

#### Behavioral biases for the analysis of individual FDM

Years later, a significant work fundamentally changed the decision theory of Raiffa (1968), contributing to the evolution of BFMI. Along with Kahneman and Riepe, 1998 wrote a paper entitled “*Aspects of Investor Psychology: Beliefs*, *Preferences*, *and Biases Investment Advisors Should Know About*.*”* Through this work, the authors categorized investors’ biases – today also known as *behavioral biases* – on three levels:

- *Biases of judgment*, which include overconfidence, optimism, hindsight, and overreaction to chance events.
- *Errors of preference*, which contain a non-linear weighting of probabilities; the tendency of people to value changes, not states; the value of profits and losses as a function; the form and attractiveness of gambles; the use of the purchase price as a reference point; narrow framing; trends related to repeated gambles and risk policies; and the adoption of short versus long views.
- *Biases associated with living with the consequences of decisions*, which give rise to regrets of omission and commission, and have implications regarding the relationship between regret and risk taking.

**Figure 3:**
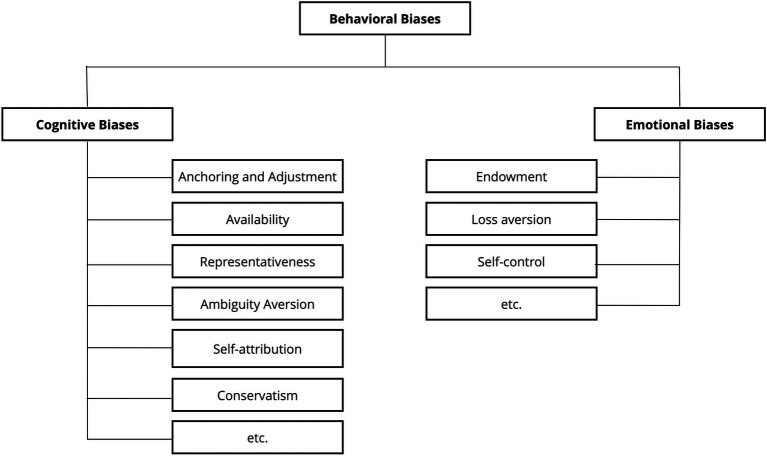
Example of behavioral biases’ taxonomy (Pompian, 2006).

Relevant research still seeks to classify behavioral biases according to some sort of meaningful framework. Some scholars refer to biases as heuristics (rules of thumb), while others mention them as judgments, beliefs, or preferences; still other authors classify biases along *cognitive* or *emotional* lines, where cognitive biases stem from faulty reasoning (such as anchoring and adjustment, availability, representativeness, ambiguity aversion, self-attribution, and conservatism) and emotional biases originate from impulse or intuition rather than conscious calculations (such as endowment, loss aversion, and self-control) (Pompian, 2006; Figure 3). It is noteworthy how, within this perspective, the term “*behavioral”* is often associated with the “*cognitive”* one; in fact, if in psychology “*mind*” and “*behavior*” assumes well disjointed meanings, in the economic language the boundary is often blurred. Similarly, the adjective “*emotional”* seems to be misused for defining what, in psychology, is termed as attitude (i.e., self-control), rather than emotion.

Researchers in the field of BFMI have distinguished a long list of specific behavioral biases, applying over 50 of these to individual investor behavior (Pompian, 2006). Indeed, several studies have been carried out to identify significant behavioral biases and investigate their influence on individual FDM, offering a great contribution in the study of how investors, with their limited resources, make decisions influenced by their previous experiences and the specific environment in which they are in. Indeed, more recent studies (Maxwell et al., 2011) have shown that angel investors use heuristic DM shortcuts known as elimination-by-aspects to reduce available investment opportunities to a more manageable dimension.

### The contribution of social psychology in the study of group FDM under risk

If the great contribution of Cognitive Psychology in Behavioral Finance focuses on FDM mainly at the individual level, some aspects that may prove to be crucial in the study of financial decisions have been addressed by Social Psychology in the investigation of *choice shift* and *group DM (GDM) under risk* (Kameda and Davis, 1990).

When it comes to GDM, the most widely studied phenomenon is that of social influence. In this regard, it is important to make a distinction between two traditional strands of research: on the one hand, the study of how the group influences the decision of the individual group member; on the other hand, how the group takes a collective decision aimed at a common goal. The first research strand has been studied for a long time by applying the functionalist paradigm of Asch (1952) favoring the influence of the majority on the behavior of individuals. This dominant perspective was then contested by Moscovici (1976) who argued the need to consider the social influence as a conflict between majority and minority that can be solved with the prevalence of the former, producing conformity, or of the second, producing innovation or, finally, with a reciprocal adaptation that gives rise to the elaboration of a norm (*normalization*). It is easy to understand how both points of view can be applied productively to the study of social influence in the area of economic and financial behavior.

The second line of research has recently acquired even more relevance since, in contemporary society, decisions are increasingly entrusted to groups – especially in the financial field – assuming that group decisions are more reliable than individual ones (Mannetti, 2004). However, given the proven evidence of complex dynamics triggered during group discussions, a question arises: is this hypothesis justified? One of the most significant phenomena investigated by the financial literature on how groups take collective decisions is known in social psychology with the expression of “*group polarization*.” This kind of social influence has been explored by Stoner (1968) his studies, finding that GDM, after group discussion, tends to be riskier than individual DM, a phenomenon that Stoner termed “*risky shift*.” Therefore, he stated that, if the initial opinions of group members tend to be risky, group decisions would be riskier (Davis, 1973; Myers and Lamm, 1976; Lilienthal and Hutchison, 1979). On the other hand, Fraser et al. (1971) found evidence also for a *cautious shift*, with groups advocating more conservative decisions than those of the individuals of the group. In other words, group discussions produce a strengthening of the prevailing initial attitudes (Moscovici and Zavalloni, 1969), a *polarization* which is supposed to be produced by both the informational and normative social influence processes (Eagly and Chaiken, 1993, p. 658).

Moreover, *group polarization* has been investigated in relation to *framing effects*^6^ that affect the group’s final decisions. Regarding this, in a study by Cheng and Chiou (2008), it was investigated whether group polarization effects reinforce framing effects. It was predicted that framing effects would be relatively stronger in GDM than in individual DM. More specifically, it was hypothesized that, after group discussions, the group polarization effect would lead decision-makers to show a lower preference for the risky option in gain situations and a greater preference for the risky option in loss situations than when they performed the investment decision task on their own. The findings of this study confirmed the hypotheses of the authors, suggesting that GDM on investments exhibits the same framing effects as individual DM, but that framing effects are more prominent in GDM situations than in individual ones (Cheng and Chiou, 2008).

### The identification of a gap in FDM theories: Missing models for IHC

Collectively, what seems to emerge from the analysis of the literature on FDM is that:

- The prevalence of studies is still unbalanced on the analysis of individual DM.
- Humans are often considered as bearers of biases and distortions.
- The majority of studies described one class of decision-makers: the investors.
- Psychology offers several models to study FDM, although the field of IHCs appears to be significantly less investigated than that of the capital market.

To understand why the existing study approach to financial decisions does not meet the requirements for the analysis of DM in IHC, it is necessary to define the main characteristics that differentiate it from other contexts of investment:

- *IHC does not involve only individual DM*. Indeed, when it comes to IHC, most of the phenomena of psychological interest, including the DM practices, are irreducible to an individual analysis. Such analysis would risk losing sight of the social process interaction and the sharing of meanings, including cultural ones, which makes it possible to explain and describe the behavior and activities of individuals in real social contexts (Mannetti, 2004). As Guerin (2003 p. 715) rightly argues, *“we cannot separate people from economic*, *social and cultural relations even if we keep them alone”* because the economic behavior of people that we want to study are in fact “formed” by these relationships.
- *IHC does not involve only GDM*. Indeed, IHCs may require the encounter of mixed individuals (i.e., an investor and a fund seeker), or multiple groups (i.e., the management team of a Venture Capital Organization and a startup team), who start from not coincident objectives. Since groups in Social Psychology are defined as *organized sets of individuals who act to achieve a common goal*, theories on GDM can only partially explain IHC phenomena.
- *IHC is not a one-sided investment*, *but a mutual investment*. In fact, if for capital market investments the only category of decision-makers is represented by investors, IHC deals with at least two classes of decision-makers: those who invest and those who seek investments, both with agency and intentionality. For example, considering the context of startup funding, venture capitalists have to decide whether to invest their sum of capital and enter a company, but, at the same time, startuppers have to decide whether to offer their resources and knowledge at the service of those venture capitalists rather than other lenders.

For those reasons, we define this kind of DM a MADM and suppose that, being a complex multilayer process, it requires a more inclusive theory that helps modeling the DM behaviors of all the actors involved in the decision process – *meaning multiple individuals who*, *starting from different objectives*, *meet each other’s to reach a mutual agreement*. We are aware that this psychological analysis of FDM can benefit from numerous insights that come from other fields, such as psychological studies of entrepreneurship. These studies explore many crucial aspects of entrepreneurs’ DM in conditions of extreme uncertainty and ambiguity, such as the moral imagination that integrates ethical dimensions (Smolka et al., 2018) or the interaction between social and psychological capital that underlies a social entrepreneurship project (Modesti et al., 2020). However, although they may constitute a complementary perspective to our analysis, it is necessary to specify that, as will be understood in the following paragraphs, our vision is focused on activity systems as a whole rather than on individual processes.

### Shared reality theory: A first model to the analysis of MADM

**Figure 4:**
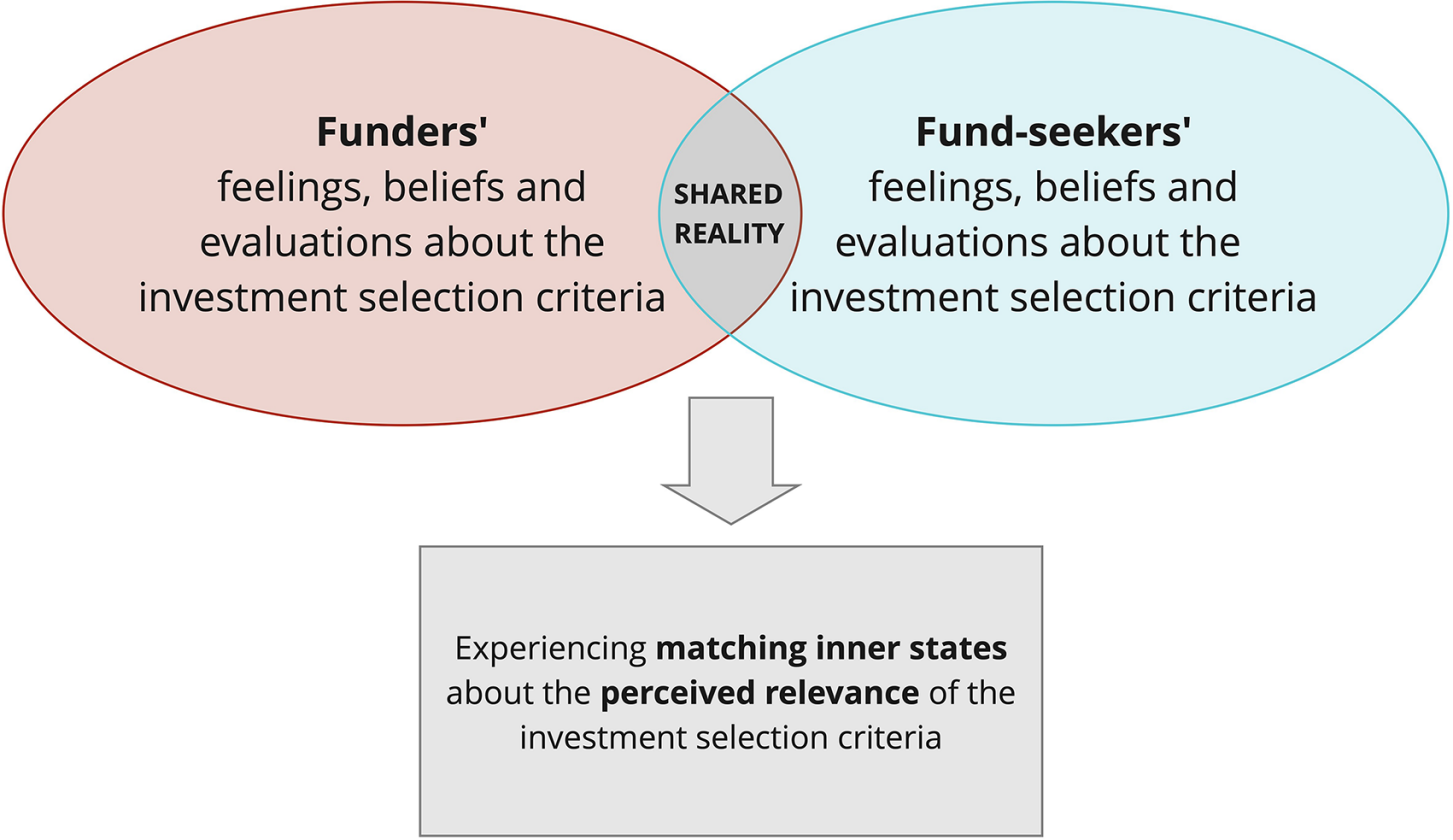
The experience of shared reality in a dialogue between funders and fund seekers.

As seen above, one of the aspects of MADM that differentiates it from GDM is the lack of necessarily shared and common objectives among the decision-makers. According to us, an interesting theory that can be adapted to the study of MADM – with the aim of favoring the sharing of meanings among decision-makers – is that of *shared reality* (Echterhoff, 2012). Precisely, according to Echterhoff and Higgins (2018), two well-known exponents of social cognition, shared reality is *the experience of having in common with others inner states about the world*, *that is the perceived relevance of something*, *as well as feelings*, *beliefs*, *or evaluation*s. As a result, the perception of inner states’ commonality with others fosters the perceived truth of those inner states and intensifies the experience of making the right decision (Higgins et al., 2020). Therefore, shared reality goes beyond the mere duplication of another person’s emotions, as in the case of emotional contagion^7^ (Neumann and Strack, 2000). In this respect, shared reality requires mechanisms that allow people to deduce the inner state of their partner (Malle and Hodges, 2005; Higgins and Pittman, 2008). According to the literature, the mechanisms most commonly used to infer the inner states of others, such as beliefs and attitudes, include *conscious reasoning*, *unconscious simulation*, *and theory of mind* (Leslie et al., 2004); *causal theories and schemata* (Heider, 1958; Malle, 1999); and *projection of one’s own inner states* (Nickerson, 2001; Keysar and Barr, 2002). Until now, the concept of shared reality has been particularly relevant to Consumer Psychology, where consumers communicate with each other from word of mouth, through channels such as forums, blogs and social media. Nevertheless, we believe that studies aimed at this scope could be of great benefit even in undiscovered fields, such as the one of IHCs. Indeed, when it comes to such decisions, both kinds of decision-makers, the investors and the fund seekers, desire to reach a profitable agreement, despite starting from not necessarily coincident goals and beliefs. Encouraging the creation of shared realities during the communication between these two classes of decision-makers, not only serves to build a common ground, which implies a shared basic knowledge of the topic of the conversation, but also to allow communication actors experiencing matching inner states about the topic of the conversation, such as the feelings, beliefs, or evaluations of something (Echterhoff and Higgins, 2018). For example, there may be a common ground between funders and fund seekers, in the sense of a shared reference, regarding the perception of investors’ selection criteria. All the actors know that the evaluation of the business plan corresponds to a selection criterion. However, this would not necessarily mean that funders and fund seekers agree on their judgments or feelings about it. In fact, they might not even have shared relevance, because funders may think the business plan is a fundamental prerequisite, but fund seekers do not (Figure 4).

Moreover, previous work has shown that people are particularly inclined to create *shared reality* with those they trust epistemically (Echterhoff and Higgins, 2017), with whom they feel connected (e.g., ingroup members; Echterhoff et al., 2005, 2008; Sinclair et al., 2005; Rossignac-Milon and Higgins, 2018; or close partners, Rossignac-Milon et al., 2020), or with a group of people with a common feeling or belief (vs. with an individual; Higgins et al., 2007; Echterhoff et al., 2017). Therefore, the absence of *trust* between the subjects of communication may represent a concrete barrier to the development of shared reality and may hinder the success of the agreement. For this reason, it is worth to master these psychological mechanisms when dealing with MADM in IHCs.

### Cultural psychology and activity theory for modeling complex MADM

If shared reality theory offers a contribution in the study of MADM mostly in terms of communication – explaining how particular mechanisms may help inferring the sharing of inner states to develop an experience of commonality -, we believe this theory could benefit from integrations with other approaches to describe all the complex components of MADM. In this regard, we assume that the study of such decision processes could really take advantage by considering some conceptualizations from Socio-Cultural Psychology and activity theory (AT) (Leont’ev, 1974, 1978; Engeström, 1987, 2001).

#### A conceptual framework to understand networks of interacting activity systems

While Cognitive Psychology studies the individual and intrapsychic processes, and Social Psychology, in particular the branch of Social-Cognition, focuses on social influence and group biases, Socio-Cultural Psychology, more specifically AT, shifts the focus of the unit of analysis not on the individual, nor on the group, but on the “*activity”* itself, understood as a finalized, transformative, and developing interaction between the actors (“*subjects*”) and the world (“*objects*”). All these aspects can also be conceptualized as meaningful *choreographies* (Talamo et al., 2017). In fact, as the anthropologist Duranti coherently affirms, “*a meaning does not exist independently of its activity; not considering this aspect means studying psychological activities that are produced by experimental situations*, *not very representative and far from real situations”* (Duranti, 1997).

From its first formulation to the present day, it is possible to identify three generations of AT (Engeström, 1999). The first generation was based on Vygotsky (1978) idea of mediation (*Subject-Artifact-Object*^8^), further developed by Leont’ev and usually sketched in the form of an activity triangle. According to Engeström (2001), the example of primordial collective hunting^9^ of Leont'ev, 1981 represented a first turn toward the social AT, since it explained the difference between individual action and collective activity. Thus, Engeström took this reference to lay the groundwork for identifying the second generation of AT, called the *“Activity System Model”* (1987, Figure 5).

**Figure 5:**
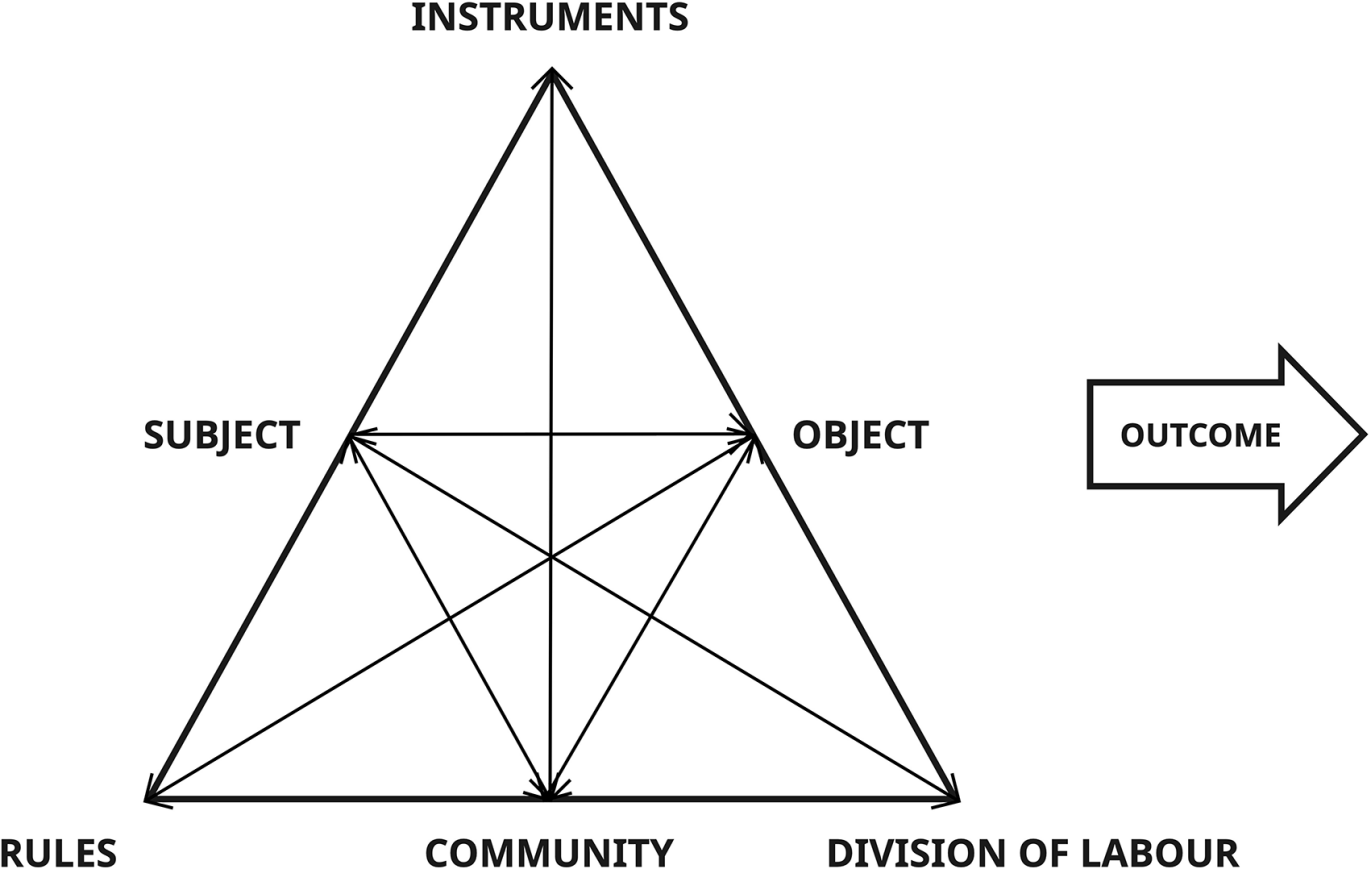
II generation of AT (Engeström, 1987).

**Figure 6:**
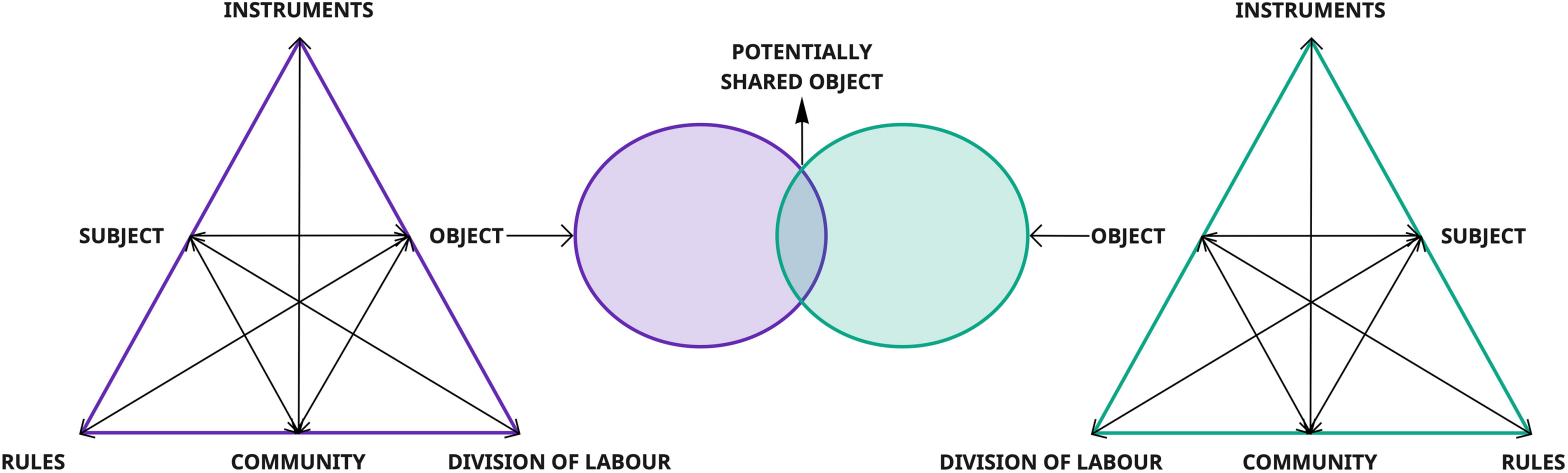
III generation of AT (Engeström, 2001).

Through the second generation of AT, Engeström expanded the *Subject-Artifact-Object* triangle, by adding three new elements of complexity. The first is *rules*: sets of conditions (formal and/or informal) that help determine how and why individuals can act and are the result of social conditioning. The second is *the division of labor* (roles and tasks), which involves the distribution of actions and operations among a community of workers. These two elements influence a new plane of reality known as *community*, through which groups of activities and teams of workers are anchored and can be analyzed (Hyland, 1998; Verenikina, 2001). Due to its social nature, the second generation of AT incorporates the idea of internal contradictions as driving forces for change and development in activity systems. This framework was further developed by the third generation of AT (Figure 6), addressing the challenge of developing *“conceptual tools to understand dialogue*, *multiple perspectives*, *and networks of interacting activity systems”* (Engeström, 2001, p. 135).

This last generation of AT is grounded on five key principles that should be taken into consideration when this framework is used to analyze complex social contexts:

- *Openness:* the main unit of analysis for research is the *artifact-mediated system of activity*, seen as part of a network that includes its relationships with other systems of activity. Therefore, *“goal-directed individual and group actions*, *as well as automatic operations*, *are relatively independent but subordinate units of analysis*, *eventually understandable only when interpreted against the background of the entire activity systems* (Engeström, 2001, p. 136).
- *Multivoicedness*: polyphony is an intrinsic property of activity systems. Therefore, activity systems are communities that incorporate multiple points of view, traditions, and interests (Engeström, 2001).
- *Historicity*: the features and the potential of activity systems can only be understood with respect to their own historical framework, since they are continuously shaped over time, along their history (Engeström, 2001).
- *Contradictions*: activities are open systems interacting with each other. Contradictions are seen as *“historically accumulating structural tensions within and between activity systems”* and therefore they constitute the major driver for change and development (Engeström, 2001, p.137).
- *Expansive transformation*: the possibility of a radical transformation within the activity systems is closely related to the afore-mentioned properties. Indeed, over time, openness and multi-voicing produce contradictions. Since contradictions are embedded in the activity of individual participants, they initiate a process of deviation from the established norms of the systems, which may trigger and deliberate a collective change in the system (Engeström, 2001).

**Figure 7:**
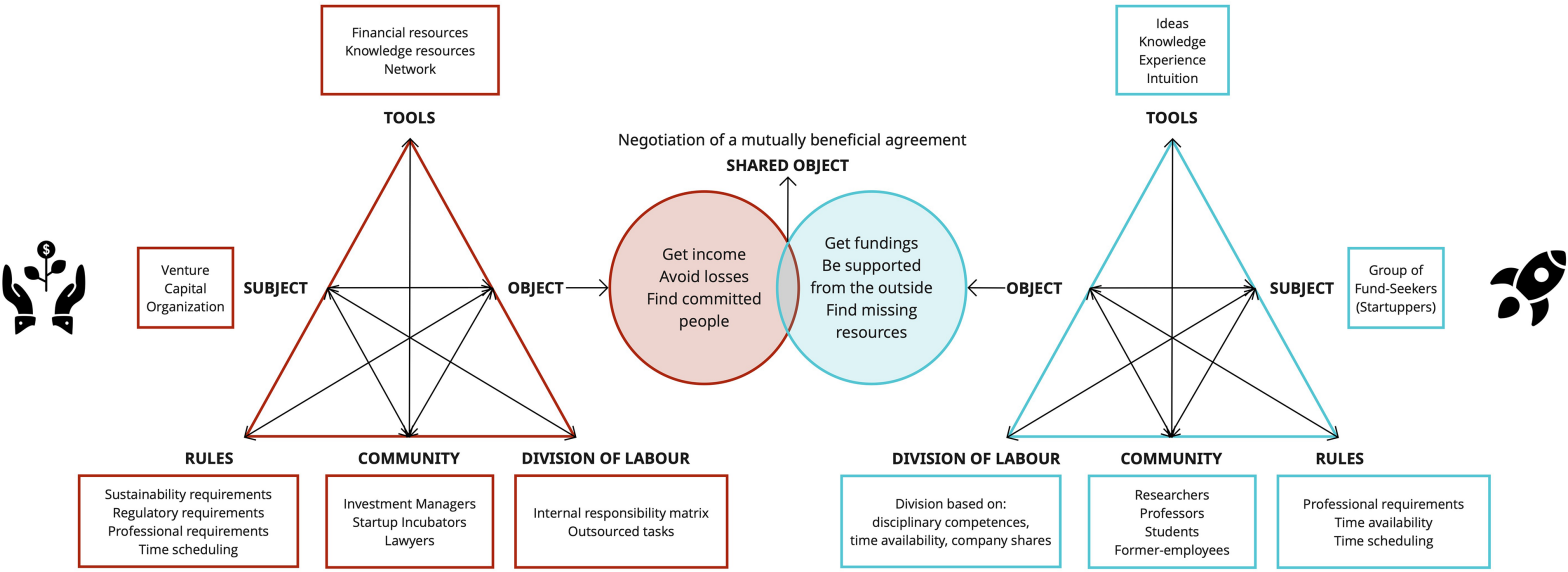
III generation of AT framed in a IHC context.

Because of its interactive and multi-voice nature, we consider the third generation of AT (Engeström, 2001) as the most appropriate model to explain the MADM construct (Figure 7). In practice, representing MADM through this model can be extremely helpful in eliciting and raising awareness of what are potential barriers and points of convergence between the involved activity systems. Especially if the creation of these diagrams comes from a specific sequence of action-research activities aimed at modeling the DM processes of different activity systems (see Talamo et al., 2021).

#### An example of multi-actor FDM: The case of startup funding

To give a concrete example, below we adapted the framework to the specific context of IHC (Figure 7), describing the case of a startup funding. Therefore, we analyzed the activity system of a Venture Capital Organization, with its own rules, tools, community, and division of labor, in interaction with the activity system of a group of fund seekers (potential startuppers), which also has its own components. Below we will go into more detail of both activity systems to understand how important it is to consider all their specific components, in order to facilitate negotiation among the decision-makers and reach a successful agreement.

Starting from the *tools*, the exchange between the activity systems takes place mainly between the offer of financial resources by the venture capitalists and the knowledge of potential startuppers. However, sometimes there may be inconsistency between the capital supply capacity of venture capitalists and the expectations of prospective founders based on their startup estimate. Also, a critical issue could arise due to the lack of specific human resources that cover functional roles to the autonomy of the startup team. Furthermore, the *communities* highlight how varied the multiplicity of target involved in the agreement can be; for instance, if on the one hand the Venture Capitalist Organization involves investment managers, lawyers, startup incubators, on the other a group of potential startuppers could be composed of researchers, professors, students, and former employees. Clearly, each of these professionals may be more or less compatible with community members belonging to the other system. For example, there may be conflicts of interest between venture capitalists, which propose their affiliated startup incubator, and an academic founder, who may have an interest in promoting a startup incubator affiliated with his Research Center. Finally, there are *rules* that investors usually propose through a document, the *term sheet*, also called a letter of intent or memorandum of understanding. This is a preliminary agreement that allows the investor to devote more time and resources to the evaluation of the investment and the startup to have already clear what will be the main clauses to be regulated later in the investment agreements. However, the negotiation of this term sheet may constitute an obstacle to the progress of the investment as it could find potential startuppers unprepared and bewildered in dictating the terms of the agreement and fully understanding the contractual clauses. Also, each activity system has its own *objectives* and expectations: if venture capitalists need to get income, avoid losses, and consequently find a group of committed and competent founders; potential startuppers, in addition to the need of receiving funding, may need support in developing the idea, creating an autonomous team or carefully defining the contractual terms. All these aspects, such as the available funds (tools), time to devote to the company, clauses to be respected (rules), convergence between objectives (objects), and contribute to creating a representation of the development prospect of the person seeking fundings. It is the comparison between this representation and the expectations and objectives of all the actors that allows the need for a negotiation among the decision-makers. In fact, we must remember that, when we deal with IHC, we refer to the development prospects of people that deliberately determine their future. Hence, we cannot overlook the will, goals, and motivations of each human actor involved in the DM process, because of their strong impact on the outcome of the DM. When expectations are not met and the objectives of the decision-makers do not find points of convergence, the deal is easily interrupted causing damage and losses not only to potential startuppers but also to investors.

#### Design thinking as bridging techniques between activity systems

If up to now we have described the hypothesis of compatibility/incompatibility of the systems between their various components, it is necessary to face a final step to understand how, in practice, this model may facilitate the creation of points of convergence and support the final DM. First, the application of this model requires an analysis of the real context in which the activity systems operate; for this reason we prompt investors and fund-seekers to rely on professionals in the field of psychology who have expertise in the design thinking^10^ approach, proposing a specific sequence of activities aimed at modeling specific phases of DM processes by different actors. In fact, design thinking combined with maieutic techniques, typical of expertise of psychologists, fosters modeling the complexity of DM emerging from different actors around funding decisions. The specific sequence we propose is composed of four principal steps (see Talamo et al., 2021):

- Enhancing venture capitalists’ DM awareness: The first step aims at producing an increased awareness in management on their own intentions and funding criteria that will support their DM.
- Exploring fund-seekers: This second step aims at studying the potential fund-seekers and their psychological world to collect data on which the modeling activity can be based.
- Modeling activities of fund-seekers and DM processes: This third step leads to a full-fledged view of the fund-seekers. The collected data would be beneficial to the venture capitalists, providing insights about the contexts, will and motivations of fund-seekers.
- Bridging funders and fund-seekers: This last step, matching DM flow of fund-seekers with that of venture capitalists, proves to be very useful in identifying problems, developing potential bridging solutions in order to facilitate the creation of points of convergence between the activity systems.

**Figure 8:**
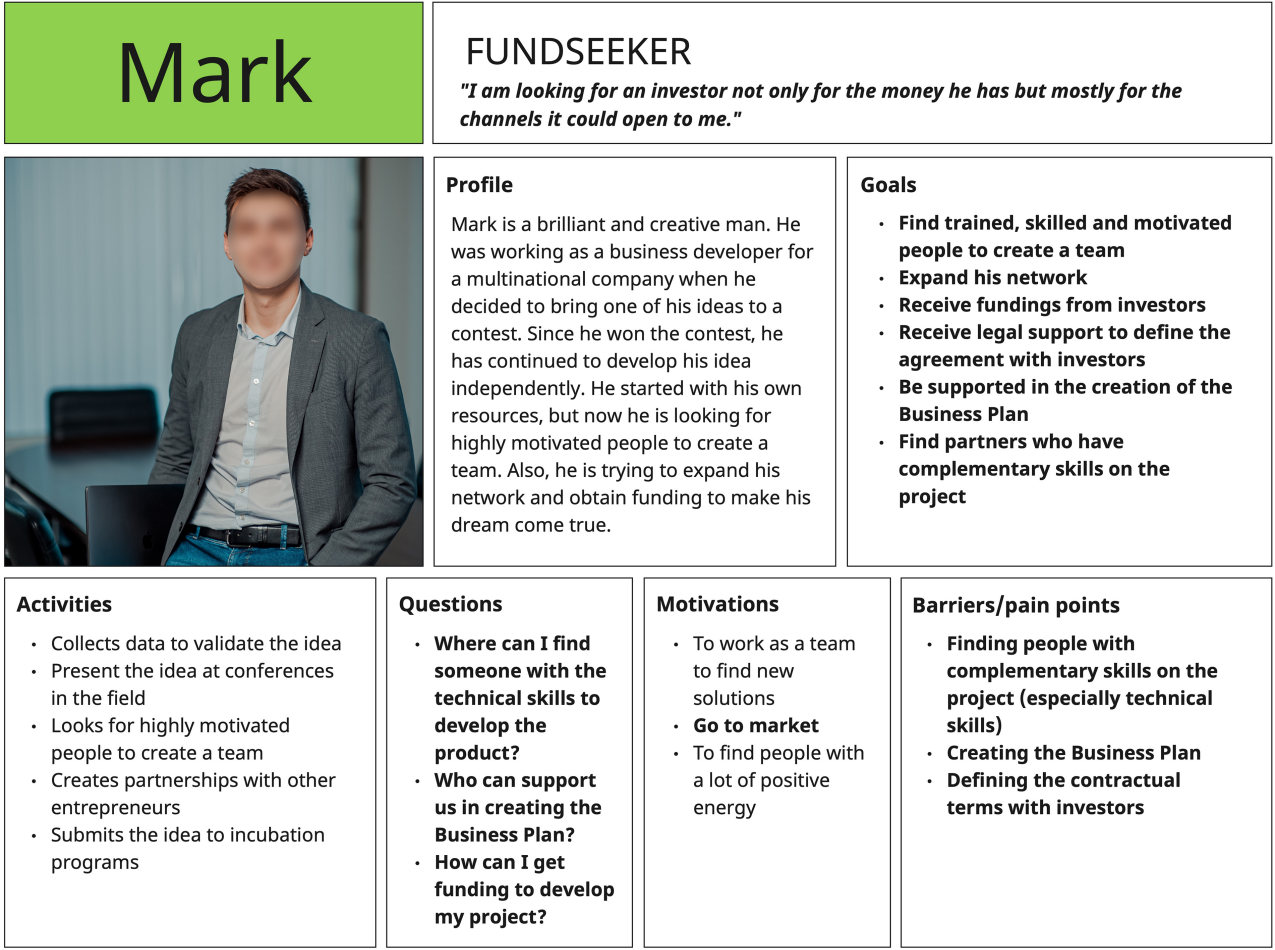
An example of *Personas* from a design thinking project in the field of startup funding.

To simplify this conceptualization, we propose a concrete example extracted from a design thinking project carried out in this area. More specifically, we will show an example of *Personas* (Figure 8)^11^ (from the target of fund-seekers) to understand how some crucial data (especially goals and barriers) become part of the activity system (*objects*) and create points of convergence within components (e.g., *tools*) of the other activity system.

In the following table (Table 1), we have reported some of the crucial goals and barriers of the Persona as *objects* of fund-seekers’ activity system and highlighted the points of convergence with other components of the venture capitalists’ activity system.

**Table 1:** Points of convergence between activity systems in the field of startup fundings.

Points of convergence	Fund-seekers	Venture capital organization
Integrate new members into the team	**Object:** Need to meet people with many contacts; need to find developers; need to find partners who have complementary skills on the project	**Possibility of development:** Network (**Tool**) and Startup Incubators (**Community**)
Get fundings	**Object**: Finding the financial capital to start	**Possibility of development:** Financial resources (**Tool**)
Receive help and support from outside	**Object**: Be supported in defining the agreement	**Barrier:** Regulatory requirements – *term sheet* (**Rules**) **Possibility of development:** Lawyers (**Community**)
Create a business model	**Object**: Be supported in creating the business plan	**Barrier:** Professional requirements – *business model* (**Rules**) **Possibility of development**: Startup Incubators (**Community**)

**Figure 9:**
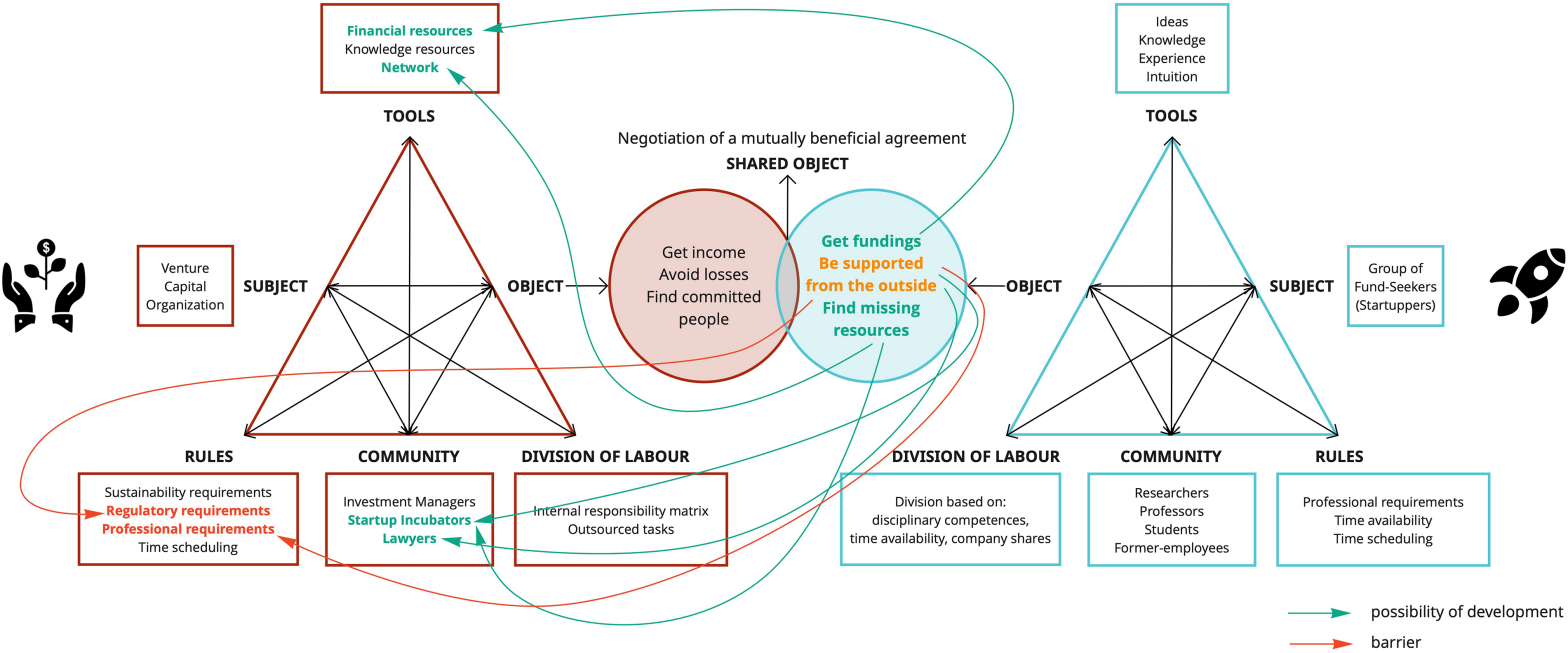
Examples of bridging points between activity systems in the field of startup fundings.

As shown in the Table 1, we started from the *objects* of the fund seekers’ activity system by analyzing different areas of experience (e.g., integrating new members into the team, creating a business model). Each *object* finds the possibility of converging with components of the other activity system reported in the Venture Capitalist column, where a barrier to overcome and/or a possibility of development is indicated. Clearly, the complementary operation can be realized starting also from the *objects* of the other activity system (that we did not report for reasons of brevity). Also below we present the diagram of activity systems in interaction (Figure 9), highlighting the above-mentioned bridging points; the possibilities for development are indicated in green and barriers in red.

Identifying points of convergence allows for starting the negotiation. Indeed, some *objects* will meet a possibility of development through the compatibility with other components of the activity system; other *objects* will require overcoming a barrier. Precisely, the negotiation will aim at overcoming these barriers in order to reach a mutually beneficial agreement. It is also important to consider the points of non-convergence as they bring out more clearly the compatibility/incompatibility characteristics of the different activity systems and the need to renegotiate some aspects of the DM. The added value of this model also derives from its visual immediacy; in fact, this visual mapping may help facilitate the elicitation of these points of convergence, raising awareness and supporting the final DM of both activity systems. For this reason, it becomes clear how important it is to map and model the components of all activity systems keeping faith with the real context in which they operate. In the end, we agree that the third generation of AT, created from this specific sequence of activities, could be extremely useful in providing theorists and practitioners with a tool to describe complex activity systems in the field of IHC, raise awareness and facilitate negotiation between different decision-makers so that their goals may become more compatible – able to coexist -, coordinable – able to complement each other – and converging – able to come closer -, in order to reach a rewarding and mutual agreement.

## Conclusion

The aim of this review is to give insight into psychological contributions to the study of FDM, especially in the field of IHC. In the first paragraphs, we reviewed the preeminent perspective of Behavioral Finance, which combines the behavioral and psychological aspects in FDM (Abay et al., 2017), identifying theories and frameworks on individual DM behaviors. Then, we focused on group DM, analyzing some phenomena of social influence such as *group polarization*, particularly relevant for financial decisions under risk. Nevertheless, we realized that neither individual and group DM theories could be supportive in describing and modeling complex social systems such as that of IHC. Indeed, IHC, differing from investment in the capital market, involves multiple *individuals who*, *starting from different objectives*, *meet each other to reach a mutual agreement*. It implies a different kind of DM process, that we call a MADM. In this view, IHC cannot be considered as a *one-sided investment*, *but a mutual investment*. In fact, if for capital market investments the only category of decision-makers is represented by investors, IHC deals with at least two classes of decision-makers: those who invest and those who seek investments, both with agency and intentionality. In this regard, the analysis of the literature showed that psychology offers several models to analyze FDM, although the field of IHCs appears significantly less investigated than that of the capital market. For this reason, we believe it is necessary to address this gap, providing a theory that may help modeling the DM of all the actors involved in the decision process. To this purpose, due to its interactive and multi-voice nature, we propose the third generation of AT (Leont’ev, 1974, 1978; Engeström, 1987, 2001) – from Socio-Cultural Psychology – as the most appropriate model to explain the MADM construct. Indeed, representing MADM processes through this model may be extremely helpful in eliciting and raising awareness of what are potential barriers and points of convergence between involved activity systems. Especially if the creation of the model comes from the use of design thinking techniques (see Talamo et al., 2021). To explain this model of MADM we used the specific example of a startup funding. Furthermore, we agree that an interesting bridge can be created between theories of different epistemologies by linking Higgins’concept of *shared reality* – which investigates what mechanisms can infer the sharing of inner states between communicators – to that of *potentially shared objects* of Engeström, so that the development of the former may increase the convergence of the second. In the end, we believe this paper may help researchers understand the gaps in the existing psychological literature on FDM and provide the scope for future work in the field of IHCs.

## Author contributions

SM and AT conceptualized the ideas presented in the article and defined the theoretical framework. SM wrote and edited the manuscript. AT supervised the whole process of writing. All authors contributed to the article and approved the submitted version.

## Conflict of interest

The authors declare that the research was conducted in the absence of any commercial or financial relationships that could be construed as a potential conflict of interest.

## Publisher’s note

All claims expressed in this article are solely those of the authors and do not necessarily represent those of their affiliated organizations, or those of the publisher, the editors and the reviewers. Any product that may be evaluated in this article, or claim that may be made by its manufacturer, is not guaranteed or endorsed by the publisher.

## Footnotes

^1^*Cognitive Psychology* is the scientific study of cognition, or the mental processes that are believed to drive human behavior. Research in cognitive psychology investigates a variety of topics, including memory, attention, perception, knowledge representation, reasoning, creativity, and problem solving.

^2^*Neoclassical Economics*: the term neoclassical economics was coined in 1900.﻿ It is based on the concept of *Homo economicus* as a simple model of human economic behavior, which assumes that principles of perfect self-interest, perfect rationality, and perfect information govern economic decisions by individuals.

^3^*Utility:* a construct in economics that measures an individual’s expressed preferences for different decision alternatives.

^4^*Bounded rationality*: the principle that organisms have limited resources, such as time, information, and cognitive capacity, with which to find solutions to the problems they face.

^5^*Loss aversion*: the tendency to react more strongly to losses than gains.

^6^*Framing effects*: Kahneman and Tversky (1979) define a framing effect as the decision-makers’ framework of reference, which is determined by their conception and by the results and contingencies associated with that particular choice.

^7^Emotional contagion: *"the tendency to mimic and synchronize automatically facial expressions*, *vocalizations*, *postures and movements with those of another person and*, *as a result*, *converge emotionally"* (Hatfield et al., 1992).

^8^*Subject-Artifact-Object:* the *subject* is the person studied, the object is the intended activity, and the *artifact* is the mediation tool with which the action is performed.

^9^*Collective primordial hunting*: an example of collective activity proposed by Leont’ev. A member of the hunting party acts as a drum beater to scare the animal. This action seems at first glance contradicting the purpose of the hunt. However, its purpose is actually to guide the animal to a place where other hunters are lurking. The action of the drum beater clearly plays a role in the realization of the general activity of the hunt. As such, it is essential that researchers reveal the true object of an activity under investigation through scientific analysis.

^10^*Design Thinking*: is the result of a multidisciplinary approach that combines methods from different disciplines to create new services or improve existing ones. It is a user-centered, co-creative and iterative approach, which uses research, prototyping and a range of activities and visualization tools to create and orchestrate experiences that meet the needs of organizations, users and other stakeholders.

^11^*Personas:* archetypes of real people of which different spheres of interest are explored, such as objectives, abilities, activities, motivations, needs and obstacles.
